# Unlocking the Transcriptomes of Two Carcinogenic Parasites, *Clonorchis sinensis* and *Opisthorchis viverrini*

**DOI:** 10.1371/journal.pntd.0000719

**Published:** 2010-06-22

**Authors:** Neil D. Young, Bronwyn E. Campbell, Ross S. Hall, Aaron R. Jex, Cinzia Cantacessi, Thewarach Laha, Woon-Mok Sohn, Banchob Sripa, Alex Loukas, Paul J. Brindley, Robin B. Gasser

**Affiliations:** 1 Department of Veterinary Science, The University of Melbourne, Werribee, Victoria, Australia; 2 Department of Parasitology, Faculty of Medicine, Khon Kaen University, Khon Kaen, Thailand; 3 Department of Parasitology and Institute of Health Sciences, School of Medicine, Gyeongsang National University, Jinju, Republic of Korea; 4 Department of Pathology, Faculty of Medicine, Khon Kaen University, Khon Kaen, Thailand; 5 Queensland Tropical Health Alliance, James Cook University, Smithfield, Cairns, Queensland, Australia; 6 Department of Microbiology, Immunology and Tropical Medicine, The George Washington University Medical Center, Washington, D. C., United States of America; University of Queensland, Australia

## Abstract

The two parasitic trematodes, *Clonorchis sinensis* and *Opisthorchis viverrini*, have a major impact on the health of tens of millions of humans throughout Asia. The greatest impact is through the malignant cancer ( = cholangiocarcinoma) that these parasites induce in chronically infected people. Therefore, both *C. sinensis* and *O. viverrini* have been classified by the World Health Organization (WHO) as Group 1 carcinogens. Despite their impact, little is known about these parasites and their interplay with the host at the molecular level. Recent advances in genomics and bioinformatics provide unique opportunities to gain improved insights into the biology of parasites as well as their relationships with their hosts at the molecular level. The present study elucidates the transcriptomes of *C. sinensis* and *O. viverrini* using a platform based on next-generation (high throughput) sequencing and advanced *in silico* analyses. From 500,000 sequences, >50,000 sequences were assembled for each species and categorized as biologically relevant based on homology searches, gene ontology and/or pathway mapping. The results of the present study could assist in defining molecules that are essential for the development, reproduction and survival of liver flukes and/or that are linked to the development of cholangiocarcinoma. This study also lays a foundation for future genomic and proteomic research of *C. sinensis* and *O. viverrini* and the cancers that they are known to induce, as well as novel intervention strategies.

## Author Summary

The parasitic worms, *Clonorchis sinensis* and *Opisthorchis viverrini*, have a serious impact on the health of tens of millions of people throughout Asia. The greatest impact, however, is through the malignant, untreatable cancer (cholangiocarcinoma) that these parasites induce in chronically infected people. These liver flukes are officially classified by the World Health Organization (WHO) as Group 1 carcinogens. In spite of their massive impact on human health, little is known about these parasites and their relationship with the host at the molecular level. Here, we provide the first detailed insight into the transcriptomes of these flukes, providing a solid foundation for all of the molecular/-omic work required to understand their biology, but, more importantly, to elucidate key aspects of the induction of cholangiocarcinoma. Although our focus has been on the parasites, the implications will extend far beyond the study of parasitic disease. Importantly, insights into the pathogenesis of the infection are likely to have major implications for the study and understanding of other cancers.

## Introduction

Liver flukes (Platyhelminthes: Digenea) include important food-borne eukaryotic pathogens of humans [1]–[5]. For example, the liver flukes *Clonorchis sinensis* and *Opisthorchis viverrini*, which cause the diseases clonorchiasis and opisthorchiasis, respectively, represent a substantial public health problem in many parts of Asia [2], [3], [6]. *Clonorchis sinensis* is endemic predominantly in regions of China (including Hong Kong and Taiwan), Korea and North Vietnam [2], [6], whilst *O. viverrini* is endemic throughout Thailand, the Lao People's Democratic Republic, Vietnam and Cambodia [7]. Both of these parasites cause immense suffering in tens of millions of people, and more than 600 million people are estimated to be at risk of infection [2], [8]. Despite efforts to control these two liver flukes, the prevalence of infection can be as high as 70% in some regions, including the Guangxi province in China (*C. sinensis*) and Khon Kaen province in Thailand (*O. viverrini*) [2], [7]. A related fluke, *O. felineus*, is endemic in Siberia and eastern regions of the former USSR, and causes a similar disease and disease burden to *O. viverrini* and *C. sinensis* [4].

The life cycles of *C. sinensis* and *O. viverrini* are similar [9]–[11], involving an aquatic snail (order Mesogastropoda), in which asexual reproduction takes place, and freshwater cyprinid fishes or palaemonid shrimps (for *C. sinensis* only) as intermediate hosts. Fish–eating ( = piscivorous) mammals, including humans, dogs and cats, act as definitive hosts, in which sexual reproduction occurs. Clonorchiasis and opisthorchiasis are prevalent in geographical regions where raw cyprinid fish (*C. sinensis* and *O. viverrini*) and/or shrimp (*C. sinensis*) are a staple of the diet of humans [2], [12]. Both parasites establish in the bile ducts of the liver as well as extrahepatic ducts and the gall bladder of the mammalian (definitive) host. These parasites are long-lived and cause chronic cholangitis, which can lead to periductal fibrosis, cholecystitis and cholelithiasis, obstructive jaundice, hepatomegaly and/or fibrosis of the periportal system [13]–[18]. Importantly, both experimental and epidemiological evidence [19]–[22] strongly implicates *C. sinensis* and *O. viverrini* infections in the etiology of cholangiocarcinoma, a malignant cancer of the bile ducts in humans which has a very poor prognosis. Indeed, *C. sinensis* and *O. viverrini* are both categorized by the International Agency for Research on Cancer (IARC) as Group 1 carcinogens [23].

In humans, the onset of cholangiocarcinoma occurs with chronic clonorchiasis or opisthorchiasis, associated with hepatobiliary damage, inflammation, periductal fibrosis and/or cellular responses to antigens from the infecting fluke [24]. These conditions predispose to cholangiocarcinoma, possibly through an enhanced susceptibility of DNA to damage by carcinogens [19], [20], [25]–[27]. Chronic hepatobiliary damage is reported to be multi-factorial and considered to arise from a continued mechanical irritation of the epithelium by the flukes present, particularly via their suckers, metabolites and excreted/secreted antigens [28], [29] as well as immunopathological processes [21]. In regions where *O. viverrini* is highly endemic, the incidence of cholangiocarcinoma is unprecedented [3], [30]. For instance, cholangiocarcinomas represent 15% of primary liver cancer worldwide, but in Thailand's Khon Kaen region, this figure escalates to 90%, the highest recorded incidence of this cancer in the world [31].

Currently, there is no effective chemotherapy to combat cholangiocarcinoma, such that intervention strategies need to rely on the prevention or treatment of liver fluke infection/disease. Although effective prevention could be readily achieved by persuading people to consume cooked fish and shrimp (*via* education programs), the ancient cultural custom to consume raw, undercooked or freshly pickled fish and shrimp persists in endemic areas [2], [3], [6]. Thus, currently, the control of clonorchiasis/opisthorchiasis relies predominantly on anthelmintic treatment with praziquantel. Despite the efficacy of this compound, the lack of an acquired immunity to infection predisposes humans to reinfections in endemic regions [2], [24]. In addition, under experimental conditions, the short-term treatment of *O. viverrini*-infected hamsters with praziquantel (400 mg per kg of live weight) has been shown to induce a dispersion of parasite antigens, resulting in adverse immunopathological changes as a result of oxidative and nitrative stresses following re-infection with *O. viverrini* [32], a process which has been proposed to initiate and/or promote the development of cholangiocarcinoma in humans [33]. Given the current reliance on a single trematocidal drug against *C. sinensis* and *O. viverrini*, there is substantial merit in searching for new intervention methods, built on a detailed understanding of the interplay between the parasites and their hosts as well as the biology of the parasites themselves at the molecular level. Furthermore, the characterization of the genes expressed in these parasites should assist in elucidating the molecular mechanisms by which clonorchiasis and opisthorchiasis (or the respective parasites) initiate and enhance the development of cholangiocarcinoma [29], [34].

To date, most molecular biological research of socioeconomically important trematodes has focused on the human blood flukes, *Schistosoma mansoni* and *S. japonicum*, recently culminating in the determination of their nuclear genome sequences [35], [36]. These genomic data sets provide an invaluable resource to support the exploration of the fundamental biology and evolution of flukes as well as their host–parasite interactions [36]. However, the biology of schistosomes, which live as dioecious adults in the blood stream of mammalian hosts, is vastly distinct from that of hermaphroditic liver flukes, such as *C. sinensis* and *O. viverrini*. Currently, a total of only ∼8,000 expressed sequence tags (ESTs) are publicly available for *C. sinensis* [37]–[39] and *O. viverrini* [40], a dataset far too small to give sufficient insights into transcriptomes for the purpose of supporting genomic and other fundamental molecular research.

Some recent genomic, bioinformatic and proteomic studies [29], [41]–[45] indicate unique and exciting prospects to explore key biochemical, physiological and biological pathways in liver flukes, and to predict and prioritize novel drug targets. In particular, the characterization of the transcriptome of the common liver fluke, *Fasciola hepatica* using next-generation sequencing-bioinformatic platform has discovered numerous molecules of biological relevance, some of which are inferred to be involved in key biological processes or pathways that could serve as key targets for new trematocidal drugs or vaccines [46]. Using a similar platform, we characterized herein the transcriptomes of the adult stages of *C. sinensis* and *O. viverrini*, in order to provide essential resources for future genomic, proteomic, metabolomic and systems biological explorations of these important pathogens, and to underpin future efforts toward the improved intervention and control of cholangiocarcinoma.

## Materials and Methods

### Production of *Clonorchis sinensis* and *Opisthorchis viverrini*

Metacercariae were collected from naturally infected cyprinoid fish, using established methods [40], [47], in the Jinju-si, Gyeongsangnam-do province, South Korea (*C. sinensis*) and the Khon Kaen province, Thailand (*O. viverrini*). Helminth-free inbred Syrian golden hamsters (*Mesocricetus auratus*) were infected with metacercariae of each species as described previously [40], [47]. Hamsters used in this study were maintained at the animal research facilities at the Faculty of Medicine, Khon Kaen University, Thailand and the School of Medicine, Gyeongsang National University, South Korea. All work was conducted in accordance with protocols approved by the animal ethics committees of respective institutions. Thirty-one (*C. sinensis*) to 42 (*O. viverrini*) days after infection, adult flukes were collected from the bile ducts of hamsters and cultured *in vitro* to allow the worms to regurgitate caecal contents using an established procedure [46]. Subsequently, all flukes were washed extensively in physiological saline, snap-frozen in liquid nitrogen and then stored at −80°C. The specific identity of the adult worms was verified by isolating genomic DNA [48] and conducting PCR-coupled, bidirectional sequencing (ABI 3730xl DNA analyzer, Applied Biosystems, California, USA) of the second internal transcribed spacer (ITS-2) of nuclear ribosomal DNA under optimized conditions [49].

### Sequencing and assembly of sequence data sets

The transcriptomes of both *C. sinensis* and *O. viverrini* were characterized by 454 sequencing (Roche) from normalized, complementary DNA (cDNA) libraries (Eurofins MWG Operon, Ebersberg, Germany; www.eurofinsdna.com) following the approach applied to *F. hepatica* [46]. For the construction of the libraries, total RNA was isolated from ∼20 adult worms of each *C. sinensis* and *O. viverrini*, and polyadenylated (polyA+) RNA was then purified from 25 µg of pooled total RNA. First-strand cDNA synthesis of polyA^+^ RNA was primed using a hybrid, random hexamer (N6) oligonucleotide containing a specifically designed adapter (5′- TCGCAGTGAGTGACAGGCCA-3′) and transcribed using M-MLV H^−^ reverse transcriptase. After RNA hydrolysis, a specifically designed adapter primer was attached to the 3′-end of the first-strand cDNA (5′-AGTCAGGACCTTGGCTGTCACTC-3′). The adapter sequences on both ends of the cDNA were then used to synthesize second-strand cDNA and amplify (18 cycles) the cDNA employing oligonucleotides complementary to the adapters by long and accurate PCR (LA-PCR) [50]. Subsequently, specific adapter sequences A (5′- CCATCTCATCCCTGCGTGTCTCCGACTCAG -3′) and B (3′- CTGAGACTGCCAAGGCACACAGGGGATAGG -5′) (FLX Titanium, Roche) were added to the 5′- and 3′-ends of the cDNA, respectively. Normalization was conducted using one cycle of denaturation and reassociation of the cDNA. Reassociated double-stranded cDNA was separated from the remaining single-stranded cDNA (ss-cDNA, normalized cDNA) by purification on a hydroxylapatite column [51]. The ss-cDNA was amplified (13 cycles) using LA-PCR and then size-selected (500–700 bp) following agarose gel electrophoresis and excision from the gel. Size-selected cDNA was eluted from the preparative gel and sequenced using a Genome Sequencer™ (GS) FLX Titanium Instrument (Roche Diagnostics) using a standard protocol [52]. The 454 Life Sciences (Roche Diagnostic) software was used for image capture and signal processing. For each transcriptomic data set, a single file containing the trace, “base-calling” and quality score data was generated and stored in a standard flowgram format (SFF) for subsequent bioinformatic processing and analyses.

An automated, *in silico*-assembly pipeline (Eurofins MWG Operon) was used to assemble *de novo* the sequence data for each *C. sinensis* and *O. viverrini*. High quality, base-called and clipped reads from each data set were extracted from the SFF-files and their contigs assembled using MIRA v.2.9 (http://chevreux.org/projects_mira.html) [53]. Mean lengths ± standard deviations in bases were calculated for particular nucleotide sequence data subsets. A second assembly of each data set was conducted using sequence regions predicted to encode open reading frames (ORFs) to specifically cluster sequences with similar protein coding regions [46]. ORFs were predicted from the MIRA-assembled contigs and -unassembled singletons using ESTScan employing default settings [54]. For each data set, sequences with ORFs were re-assembled into supercontigs using the Contig Assembly Program v.3 (CAP3) [55]. To remove redundancy, nucleotide sequences were re-clustered using the BLASTclust program (BLAST v.2.2.20; ftp://ftp.ncbi.nlm.nih.gov/blast/executables/), allowing sequences to cluster if they aligned across >60% of their length and shared >95% amino acid residue identity.

### Annotation

The transcriptome data sets for *C. sinensis* and *O. viverrini* were each annotated using a semi-automated bioinformatic pipeline [46] using stringent statistical criteria. In brief, sequences were subjected to BLASTn (searching for gene homology) and BLASTx (searching for protein homology) analyses against publicly available (December, 2009) sequences from GenBank (National Center for Biotechnology Information; http://www.ncbi.nlm.nih.gov/est/) for *C. sinensis* (n = 2,970), *O. viverrini* (4,194) and non-redundant sequence databases; ENSEMBL (http://www.ensembl.org/); SchistoDB (http://schistodb.net/schistodb20/) for *S. mansoni*; and the Shanghai Centre for Life Science & Biotechnology Information (http://lifecenter.sgst.cn/sjapathdb/data.html) for *S. japonicum* as well as a transcriptomic data set available for *F. hepatica* [46] using permissive (E-value: <1E^−05^), moderate (<1E^−15^) and/or stringent (<1E^−30^) search strategies. Homologues were identified in other eukaryotic organisms using permissive, moderate and stringent search strategies. ORFs were predicted from the final transcriptomic data sets for *C. sinensis* and *O. viverrini* using ESTScan, and proteins were inferred from ORFs by conceptual translation. Predicted proteins were classified functionally using InterProScan [56], employing the default search parameters. Based on their homology to conserved domains and protein families, predicted proteins of *C. sinensis* and *O. viverrini* were individually classified according Gene Ontology (GO) categories and assigned parental (i.e. level 2) terms (http://www.geneontology.org/). Inferred proteins with homologues in organisms for which sequence data were available were subjected to analysis, utilizing KEGG-Orthology Based Annotation System (KOBAS) [57], which predicts the biochemical pathways in which molecules are involved.

Amino acid sequences were subjected to analysis using TMHMM (a membrane topology prediction program) [58] to predict transmembrane domains. Putative excretory/secretory (ES) proteins were predicted from inferred amino acid sequences representing *C. sinensis* and *O. viverrini* using a previously described bioinformatic pipeline [44]. Briefly, ES proteins were selected based on the presence of a signal peptide at the N-terminus using SignalP 3.0 [59] and the absence of transmembrane domains. To provide further support for their classification, predicted ES proteins of >50 amino acid residues in length were compared with known secreted proteins [60] and signal peptides [61] (http://www.signalpeptide.de/), and the subset of proteins with known homologues (BLASTn, E-value<1E^−05^) were retained and summarized based on the biochemical pathway inferred using KOBAS.

## Results

### Assembly of the transcriptomes of the adult stages of *Clonorchis sinensis* and *Opisthorchis viverrini*

More than 500,000 sequences were generated for each *C. sinensis* (n = 574,448; 351±141 bases; i.e., mean ± standard deviation) and *O. viverrini* (642,918; 373±133 bases) (Table 1). Sequence data were deposited under accession number SRA012272 in the sequence read archive of NCBI (http://www.ncbi.nlm.nih.gov/sra). BLASTn searches (E-value<1E^−05^) revealed that most (92–97%) of sequences available in public databases for these flukes were contained within their respective data sets. As most (88–91%) sequences generated for each species were novel, only the present data were assembled (see Table 1). The assembly allowed ∼84% of sequences to be clustered into >42,000 contigs. For *C. sinensis*, 42,179 contigs were 711±483 bases in length, with a mean depth of coverage of 10.8±20.0 reads per contig. For *O. viverrini*, 60,833 contigs were 680±438 bases in length, with a mean depth of coverage of 8.6±14.5 reads per contig. Total numbers of 92,123 (279±161 bases; *C. sinensis*) and 101,654 (307±162 bases; *O. viverrini*) sequences were singletons and could thus not be assembled.

**Table 1:** Summary of the clustering performance and bioinformatic analyses performed on the nucleotide sequences encoded in the transcriptome of the adult stage of each *Clonorchis sinensis* and *Opisthorchis viverrini*.

	cDNA libraries	
Initial clustering	*Clonorchis sinensis*	*Opisthorchis viverrini*
Sequences before clustering	574,448	642,918
	(351±141; 1–727)^a^	(373±133; 1–724)
Proportion of sequences incorporated into clusters	83.96% (482,325)^b^	84.19% (541,264)
Contigs	42,179	60,833
	(711±483; 42–11,947)	(680±438; 41–9,753)
Singletons	92,123	101,654
	(279±161; 40–727)	(307±162; 40–724)
Total unique sequences after assembly	134,302	162,487
	(415±363; 40–11,947)	(447±348; 40–9,753)
Coverage (average reads per assembled contig)	10.8±20.0	8.6±14.5
Containing an open reading frame (ORF)	88,714 (66.1%)	107,217 (66.0%)

Re-clustering (ORF-enriched sequences)		
Sequences before clustering	88,714	107,217
	(383±371; 51–11,874)	(389±355; 51–9,753)
Proportion of sequences incorporated into clusters	44,557 (50.2%)	53,546 (49.9%)
Supercontigs	12,050	14,698
	(981±747; 51–13,604)	(939±731; 51–12,044)
Contigs	15,364	21,959
	(418±282; 51–4,161)	(390±270; 51–4,281)
Singletons	28,793	31,712
	(237±133; 51–537)	(246±134; 51–552)
Total unique sequences after assembly	56,207	68,369
	(446±484; 51–13,604)	(441±467; 51–12,044)
Coverage (average reads per assembled contig)	3.7±2.6	3.6±2.9
Possible host or other contaminants quarantined	1,578 (2.8%)	1,793 (2.6%)

a Summarized as number of sequences (average sequence length ± standard deviation; minimum and maximum sequence lengths).

b Summarized as number of sequences (proportion of total sequences used for the analysis).

In total, 134,301 *C. sinensis* sequences (415±363 bases) and 162,487 *O. viverrini* sequences (447±348 bases) were retained for further analyses. From the MIRA-assembled data, ORFs were predicted for 88,714 (66.1%) of *C. sinensis* sequences (383±371 bases) and 107,217 (66.0%) of *O. viverrini* sequences (389±355 bases). CAP3 clustered approximately half of these ORFs into ORF-enriched supercontigs, equating to 12,050 sequences (980±747 bases) for *C. sinensis* and 14,698 sequences (939±731 bases) for *O. viverrini*, with an average depth of coverage of 3.6–3.7 reads per supercontig for each species. For each species, the average G+C content (∼47±4%) was similar to the estimates for *F. hepatica*, a digenean trematode related to *C. sinensis* and *O. viverrini* [46], [62]. From either data set, a small number (49–82) of redundant sequences were excluded following the re-clustering of the sequences using BLASTclust. In addition, sequences with similarity at the nucleotide (E-value<1E^−05^) and protein (E-value<1E^−50^) levels to potential host (*M. auratus*) molecules or microbial organisms were excluded. The ORFs of both clustered and unique sequences (singletons) were subjected to further analysis.

### Annotation of proteins encoded in the transcriptome of *Clonorchis sinensis* and *Opisthorchis viverrini*

The transcriptomic data sets for *C. sinensis* and *O. viverrini* were each used to interrogate genomic databases (i.e. *F. hepatica*, NCBI non-redundant, *S. mansoni* and *S. japonicum* databases) using BLASTx (Table 2). Of the ORF-enriched sequences, 16,892 of 50,769 (33.3%) *C. sinensis* and 19,047 of 61,417 (31.0%) *O. viverrini* sequences matched known proteins at a cut-off value of <1E^−05^ (Table 2). Proteins inferred for each *C. sinensis* and *O. viverrini* were compared specifically with one another and with complete proteomic data sets for selected organisms, (i) *Saccharomyces cerevisiae* (yeast) (ii) *F. hepatica*, *S. mansoni* and *S. japonicum* (trematodes) (iii) *Caenorhabditis elegans* (nematode) (iv) *Drosophila melanogaster* (insect), (v) *Danio rerio*, *Gallus gallus*, *Xenopus tropicalis* (non-mammalian vertebrates), and (vi) *Homo sapiens* and *Mus musculus* (mammals) (Table 3). Proteins predicted for *C. sinensis* (n = 50,769) and *O. viverrini* (61,417) had the highest homology to one another using the permissive (27,103–29,995 sequence matches, equating to 48.4–53.4%), moderate (21,036–22,216 matches; 36.2–41.4%) and stringent (15,769–16,324 matches; 26.6–31.1%) search strategies. Both *C. sinensis* and *O. viverrini* shared greatest amino acid sequence similarity to proteins of other members of the Trematoda considered here, resulting in 14,526–27,103 sequence matches (28.6–53.4%) for the former and in 15,982–29,995 matches (26.0–48.8%) for the latter species (at E-value <1E^−05^). In agreement with the data available for *Schistosoma* spp. [35], [36] and *F. hepatica* [46], both *C. sinensis* and *O. viverrini* shared greater amino acid sequence similarity (E-value: <1E^−05^) to mammalian proteins [with 10,164–11,238 sequence matches (18.3–20.1%)] than to those of *C. elegans* [with 8,029–8,951 sequence matches (14.6–15.8%)].

**Table 2:** Summary of the bioinformatic analyses performed on the amino acid sequences encoded by the transcriptome of the adult stage of *Clonorchis sinensis* and *Opisthorchis viverrini*.

	cDNA libraries	
Characterization of transcripts	*Clonorchis sinensis*	*Opisthorchis viverrini*
Nucleotide sequences containing a predicted ORF	50,769 (92.9%)^a^	61,417 (92.3%)
Full-length transcripts (containing start and stop codons)	3,113	4,144
Partial transcripts with start codon only	8,466	11,407
Partial transcripts with stop codon only	10,558	13,296
Sequences with signal peptides	3,305 (6.5%)	4,246 (6.9%)
Containing transmembrane-domains	3,453 (6.8%)	4,382 (7.1%)
Putative excretory/secretory proteins	1,143 (2.3%)	1,470 (2.4%)

Homology searches		
Sequences with BLASTx hit (nr+*Schistosoma mansoni* and *S. japonicum*+*Fasciola hepatica*)	16,892 (33.3%)	19,047 (31.0%)
KOBAS (pathway mapping) results	9,847 total	11,092 total
	(4,581 sequences, 242 KEGG terms)	(5,139 sequences, 249 KEGG terms)
Coding domain match	20,369 total	22,837 total
	(9,564 sequences, 3,932 InterPro domain terms)	(11,004 sequences, 4,047 InterPro domain terms)
Gene Ontology results	18,784 total	21,400 total
	(6,580 sequences, 1,250 GO terms)	(7,586 sequences, 1,271 GO terms)

a Summarized as number of sequences (proportion of total sequences used in the analysis).

**Table 3:** Comparative genomic analysis between *Clonorchis sinensis*, *Opisthorchis viverrini*, other parasitic trematodes and selected eukaryotic (model) organisms.

	*Clonorchis sinensis* sequences (n = 50,769)			*Opisthorchis viverrini* sequences (n = 61,417)		
	with homology (%)			with homology (%)		
Predicted proteins similar^a^ to those in:	<1E^−05^	<1E^−15^	<1E^−30^	<1E^−05^	<1E^−15^	<1E^−30^
* Clonorchis sinensis*				29,995 (48.84)	22,216 (36.17)	16,324 (26.58)
*Opisthorchis viverrini*	27,103 (53.38)	21,036 (41.43)	15,796 (31.11)			
NCBI non-redundant database	16,782 (33.06)	12,164 (23.96)	7,974 (15.71)	19,126 (31.14)	13,664 (22.25)	9,093 (14.81)
* Fasciola hepatica*	15,725 (30.97)	10,580 (20.84)	6,700 (13.20)	17,495 (28.49)	11,572 (18.84)	7,186 (11.70)
* Schistosoma mansoni*	15,229 (30.00)	11,033 (21.73)	7,465 (14.70)	16,736 (27.25)	11,897 (19.37)	8,095 (13.18)
* Schistosoma japonicum*	14,526 (28.61)	10,159 (20.01)	6,429 (12.66)	15,982 (26.02)	11,116 (18.10)	7,083 (11.53)
* Mus musculus*	10,177 (20.05)	6,628 (13.06)	3,890 (7.66)	11,238 (18.30)	7,246 (11.80)	4,203 (6.84)
* Homo sapiens*	10,164 (20.02)	6,591 (12.98)	3,890 (7.66)	11,206 (18.25)	7,259 (11.82)	4,213 (6.86)
* Danio rerio*	10,000 (19.70)	6,386 (12.58)	3,744 (7.37)	11,100 (18.07)	7,043 (11.47)	4,042 (6.58)
* Gallus gallus*	9,737 (19.18)	6,187 (12.19)	3,613 (7.12)	10,718 (17.45)	6,795 (11.06)	3,889 (6.33)
* Xenopus tropicalis*	9,642 (18.99)	6,105 (12.03)	3,505 (6.90)	10,716 (17.45)	6,683 (10.88)	3,823 (6.22)
* Drosophila melanogaster*	9,032 (17.79)	5,676 (11.18)	3,292 (6.48)	10,092 (16.43)	6,251 (10.18)	3,583 (5.83)
* Caenorhabditis elegans*	8,029 (15.81)	4,847 (9.55)	2,771 (5.46)	8,951 (14.57)	5,367 (8.74)	2,974 (4.84)
* Saccharomyces cerevisiae*	4,509 (8.88)	2,371 (4.67)	1,266 (2.49)	5,194 (8.46)	2,655 (4.32)	1,397 (2.27)

a All amino acid sequences conceptually translated from ORF-enriched sequence data were searched against protein databases using BLASTx employing permissive (E-value of <1E^−05^), moderate (E-value of <1E^−15^) and stringent (E-value of <1E^−30^) search strategies.

**Figure 1:**
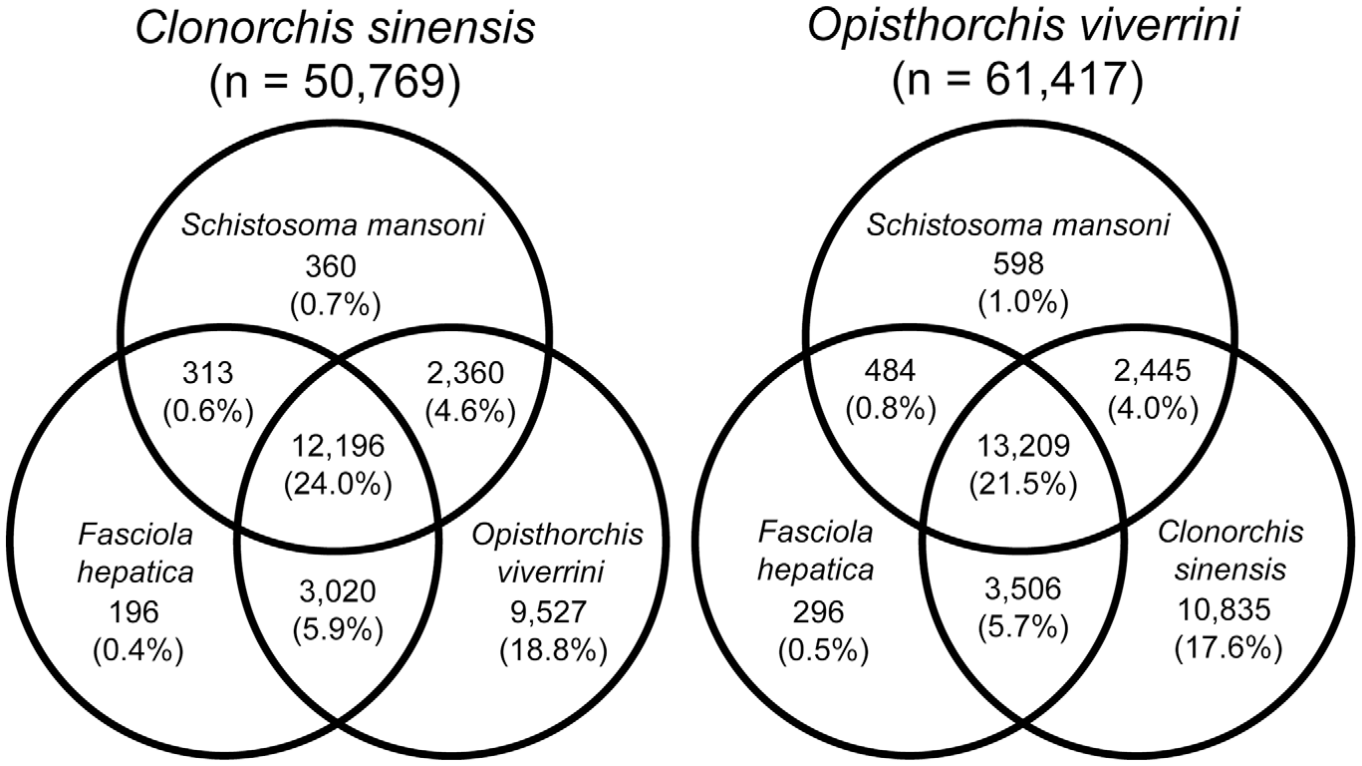
Venn diagram illustrating the overlap in sequence homology among parasitic trematodes. Predicted proteins with significant sequence similarity (permissive BLASTx search with E-value <1E^−05^) among parasitic trematodes, *Clonorchis sinensis* and *Opisthorchis viverrini* (family Opisthorchiidae), *Schistosoma mansoni* (Schistosomatidae) and *Fasciola hepatica* (Fasciolidae).

Within the class Trematoda, a high degree of protein sequence homology (21.5–24%) was shared (E-value <1E^−05^) amongst representatives of the families Fasciolidae (*F. hepatica*), Schistosomatidae (*S. mansoni*) and Opisthorchiidae (*C. sinensis* or *O. viverrini*) (Fig. 1). More proteins (9,527–10,835 matches; 17.6–18.8%) were uniquely shared between the two members of the family Opisthorchiidae than among representatives of different families. Protein conservation was also evident when *C. sinensis* and *O. viverrini* data sets were compared with the other trematodes included herein, using permissive (10,875–11,780 matches; 19.2–21.4%), moderate (7,164–7,660; 12.5–14.1%) and stringent (4,320–4,529; 7.4–8.5%) search strategies (Table 4). Relative conservation of inferred proteins was observed also when the *C. sinensis* and *O. viverrini* data sets were compared with those for mammals (mouse or human); 9,954–10,983 sequences (18–19.6%) had significant matches (E-value <1E^−05^). A significant percentage (6.1–6.8%; E-value <1E^−05^) of the proteins predicted for the two Asian liver flukes were conserved across the eukaryotic model organisms considered. These molecules included actin-like proteins, alpha and beta-tubulins, dynein-1-alpha heavy chain, elongation factor EF-2, enolase, glycogen synthase 1, heat shock protein 70, nucleosome assembly protein 1-like protein and ubiquitin-activating enzyme E1 (E-value <1E^−100^), of which most sequence matches (72.3–83.2%; E-value <1E^−05^) were to proteins inferred for *S. cerevisiae* (Table 3).

**Table 4:** Comparative genomic analysis between or among *Clonorchis sinensis, Opisthorchis viverrini*, *Fasciola hepatica*, *Schistosoma mansoni*, *S. japonicum* (blood flukes) and selected mammals.

	*Clonorchis sinensis* sequences			*Opisthorchis viverrini* sequences		
	(n = 50,769) with homology (%)			(n = 61,417) with homology (%)		
Proteins predicted to be similar^a^ to those in:	1E^−05^	1E^−15^	1E^−30^	1E^−05^	1E^−15^	1E^−30^
* S. japonicum* and *S. mansoni*	13,254	9,325	5,940	14,438	10,067	6,459
	(26.10)	(18.37)	(11.70)	(23.51)	(16.39)	(10.52)
* F. hepatica* and *S. japonicum* and *S. mansoni*	11,134	7,403	4,508	12,167	7,994	4,762
	(21.93)	(14.58)	(8.88)	(19.81)	(13.02)	(7.75)
* O. viverrini* and *F. hepatica and S. japonicum* and *S. mansoni*	10,875	7,164	4,320			
	(21.42)	(14.11)	(8.51)			
* C. sinensis* and *F. hepatica and S. japonicum* and *S. mansoni*				11,780	7,660	4,529
				(19.18)	(12.47)	(7.37)
* Homo sapiens* and *Mus musculus*	9,954	6,475	3,829	10,983	7,116	4,143
	(19.61)	(12.75)	(7.54)	(17.88)	(11.59)	(6.75)
Eukaryotic model organisms^b^	3,434	1,732	906	3,753	1,856	947
	(6.76)	(3.41)	(1.78)	(6.11)	(3.02)	(1.54)

a ORF-enriched sequence data were searched against protein databases by BLASTx using permissive (E-value of <1E^−05^), moderate (E-value of <1E^−15^) and stringent (E-value of <1E^−30^) search strategies.

b Proteins that were homologous to model organisms assessed in Table 3.

When sequences of *C. sinensis* and *O. viverrini* with homology to those within non-redundant gene data sets (available from the *S. mansoni*, *S. japonicum* and ENSEMBL gene databases) were clustered (BLASTx, E-value <1E^−05^), the number of homologous sequences predicted to encode proteins was 1.4 to 2.4-fold greater than expected (see Table 5). The clustering of ORF-enriched sequences to unique genes resulted in a prediction of 22,824–31,054 genes for *C. sinensis*, and 25,871–42,692 for *O. viverrini*.

**Table 5:** Summary of the numbers of unique genes predicted to be expressed by the adult stage of each *Clonorchis sinensis* and *Opisthorchis viverrini* based on amino acid sequence similarity to model eukaryotic organisms.

*Clonorchis sinensis* predicted proteins (50,769) similar^a^ to those in:	Sequences with homology to unique genes	Cluster size^b^	Estimated number of genes^c^
* Schistosoma japonicum*	7,154	2.03±3.64 (1–208)	25,007
* Schistosoma mansoni*	6,845	2.22±2.95 (1–122)	22,824
* Danio rerio*	6,110	1.63±1.24 (1–32)	31,054
* Homo sapiens*	5,920	1.72±1.34 (1–26)	29,585
* Mus musculus*	5,872	1.73±1.34 (1–30)	29,307
* Xenopus tropicalis*	5,468	1.76±1.45 (1–33)	28,800
* Gallus gallus*	5,289	1.84±1.47 (1–24)	27,580
* Drosophila melanogaster*	4,525	1.99±1.79 (1–26)	25,452
* Caenorhabditis elegans*	4,033	1.99±2.09 (1–62)	25,511
* Saccharomyces cerevisiae*	2,060	2.19±2.89 (1–65)	23,205

*Opisthorchis viverrini* predicted proteins (61,417) similar^a^ to:			
* Homo sapiens*	7,784	1.44±1.26 (1–68)	42,692
* Schistosoma japonicum*	7,422	2.15±3.94 (1–186)	28,524
* Schistosoma mansoni*	7,049	2.37±3.27 (1–113)	25,871
* Danio rerio*	6,378	1.74±1.74 (1–85)	35,315
* Mus musculus*	6,151	1.83±1.91 (1–97)	33,634
* Xenopus tropicalis*	5,683	1.88±2.12 (1–95)	32,589
* Gallus gallus*	5,471	1.96±2.05 (1–98)	31,365
* Drosophila melanogaster*	4,702	2.15±2.33 (1–65)	28,621
* Caenorhabditis elegans*	4,169	2.15±2.75 (1–96)	28,621
* Saccharomyces cerevisiae*	2,188	2.37±3.91 (1–100)	25,882

a All amino acid sequences conceptually translated from ORF-enriched sequence data were searched against protein databases using BLASTx employing permissive (E-value of <1E^−05^) search strategies.

b Cluster size denotes the average number of sequences (± standard deviation) clustered with a unique gene. The numbers (range) of sequences representing each cluster are given in parentheses.

c The estimated number of unique genes is based on the multiplication of the number of ORF-enriched sequences by the predicted proportion of unique genes.

To establish whether transcriptomic data sets were representative of adult *C. sinensis* and *O. viverrini*, predicted proteins were summarized according to their inferred molecular function, cellular localization and association with biological pathways (Table 2). A significant proportion (∼18–19%) of the *C. sinensis* and *O. viverrini* transcriptome was annotated using ∼4,000 unique InterPro domain or protein family signatures. Based on their annotation, according to conserved motifs, 1,250 and 1,271 different GO categories could be defined for *C. sinensis* and *O. viverrini*, respectively. All parental (i.e. level 2) GO terms assigned to the data sets for each *F. hepatica* [46] and *S. mansoni* (http://amigo.geneontology.org/; http://schistodb.net/schistodb20/) were represented in the transcriptomic data sets of the two Asian flukes (Table 6), including 19 linked to ‘biological process’, eight to ‘cellular component’ and 13 to ‘molecular function’ terms. The GO profiles were similar between *C. sinensis* and *O. viverrini*, with only two molecular function terms, namely metallo-chaperone activity and auxiliary transport protein activity being unique to each respective data set. Predicted proteins assigned to the term ‘biological process’ were associated predominantly with: (i) cellular processes (35–36%), such as protein amino acid phosphorylation, translation and regulation of transcription; (ii) metabolic processes (33–34%), such as proteolysis, carbohydrate metabolic process and oxidation reduction; and, (iii) biological regulation processes (8%), such as regulation of transcription and signal transduction (Table 6). Proteins assigned to the term ‘molecular function’ were mainly linked to: (i) the binding of ATP, zinc ion and protein (48–49%); (ii) catalytic activities (39%) of enzymes, including protein kinases and oxidoreductases; and, (iii) transporter activity (5%), including ATPase and amino acid transmembrane transporter activity and hydrolase activity catalyzing transmembrane movement of substances (Table 6). Predicted proteins were also mapped according to cellular components such as: (i) intracellular locations (60–62%), including the nucleus, membrane, cytoplasm, ribosome and microtubule; (ii) organelles (21–22%), including the nucleus, ribosome, microtubule, microtubule associated complex and cytoskeleton; and, (iii) macromolecular complexes (13–13.7%), including the ribosome, microtubule associated complex, dynein complex, membrane coat and the nucleosome (Table 6).

**Table 6:** Functions predicted for proteins encoded in the transcriptome of the adult stage of each *Clonorchis sinensis* and *Opisthorchis viverrini* based on gene ontology (GO).

Parental GO terms	*C. sinensis* sequences (%)^a^	*O. viverrini* sequences (%)	Top GO term for *C. sinensis* (CS) and *O. viverrini* (OV)
Biological process GO:0008150			
Anatomical structure formation GO:0010926	134 (1.26)	124 (1.02)	Protein polymerization GO:0051258 (CS:56; OV:37)^b^
Biological adhesion GO:0022610	76 (0.72)	100 (0.83)	Homophilic cell adhesion GO:0007156 (CS:44; OV:65)
Biological regulation GO:0065007	829 (7.82)	952 (7.86)	Regulation of transcription, DNA-dependent GO:0006355 (CS:138;OV:163)
Cellular component biogenesis GO:0044085	164 (1.55)	155 (1.28)	Protein polymerization GO:0051258 (CS:56; OV:37)
Cellular component organization GO:0016043	243 (2.29)	247 (2.04)	Protein polymerization GO:0051258 (CS:56; OV:37)
Cellular process GO:0009987	3786 (35.70)	4254 (35.13)	Protein amino acid phosphorylation GO:0006468 (CS:321; OV:345)
Death GO:0016265	10 (0.09)	17 (0.14)	Regulation of apoptosis GO:0042981 (CS:6; OV:11)
Developmental process GO:0032502	27 (0.25)	40 (0.33)	Multicellular organismal development GO:0007275 (CS:18; OV:21)
Growth GO:0040007	1 (0.01)	1 (0.01)	Regulation of cell growth GO:0001558 (CS:1; OV:1)
Immune system process GO:0002376	4 (0.04)	3 (0.02)	Immune response GO:0006955 (CS:3; OV:2)
Localization GO:0051179	852 (8.03)	994 (8.21)	Transport GO:0006810 (CS:204; OV:236)
Locomotion GO:0040011	2 (0.02)	3 (0.02)	Ciliary or flagellar motility GO:0001539 (CS:1; OV:2)
Metabolic process GO:0008152	3516 (33.15)	4113 (33.96)	Protein amino acid phosphorylation GO:0006468 (CS:321; OV:345)
Multicellular organismal process GO:0032501	28 (0.26)	37 (0.31)	Multicellular organismal development GO:0007275 (CS:18; OV:21)
Multi-organism process GO:0051704	1 (0.01)	1 (0.01)	Pathogenesis GO:0009405 (CS:1; OV:1)
Regulation of biological process GO:0050789	805 (7.59)	920 (7.60)	Regulation of transcription, DNA-dependent GO:0006355 (CS:138; OV:163)
Reproduction GO:0000003	3 (0.03)	8 (0.07)	Spermatogenesis GO:0007283 (CS:2; OV:1)
Response to stimulus GO:0050896	123 (1.16)	140 (1.16)	DNA repair GO:0006281 (CS:49; OV:51)
Viral reproduction GO:0016032	1 (0.01)	1 (0.01)	Viral genome replication GO:0019079 (CS:1), viral transcription GO:0019083 (OV:1)
Cellular component GO:0005575			
Cell GO:0005623	2953 (60.19)	3393 (61.55)	Intracellular GO:0005622 (CS:640; OV:707),
Envelope GO:0031975	59 (1.20)	66 (1.20)	Nuclear pore GO:0005643 (CS:18; OV:18)
Extracellular region GO:0005576	85 (1.73)	110 (2.00)	Proteinaceous extracellular matrix GO:0005578 (CS:12; OV:13)
Macromolecular complex GO:0032991	671 (13.68)	705 (12.79)	Ribosome GO:0005840 (CS:128; OV:141)
Membrane-enclosed lumen GO:0031974	58 (1.18)	59 (1.07)	Mediator complex GO:0000119 (CS:14; OV:14)
Organelle GO:0043226	1057 (21.55)	1160 (21.04)	Nucleus GO:0005634 (CS:368; OV:413)
Synapse GO:0045202	22 (0.45)	19 (0.34)	Postsynaptic membrane GO:0045211 (CS:20; OV:17)
Virion GO:0019012	1 (0.02)	1 (0.02)	Viral capsid GO:0019028 (CS:1) viral nucleocapsid GO:0019013 (OV:1)
Molecular function GO:0003674			
Antioxidant activity GO:0016209	10 (0.09)	16 (0.13)	Glutathione peroxidase activity GO:0004602 (CS:4; OV:6)
Auxiliary transport protein activity GO:0015457		1 (0.01)	Sodium channel inhibitor activity GO:0019871 (OV:1)
Binding GO:0005488	5160 (48.56)	5757 (47.81)	ATP binding GO:0005524 (CS:919; OV:1012)
Catalytic activity GO:0003824	4159 (39.14)	4733 (39.30)	Protein kinase activity GO:0004672 (CS:289; OV:316)
Electron carrier activity GO:0009055	68 (0.64)	96 (0.80)	Electron carrier activity GO:0009055 (CS:68; OV:96)
Enzyme regulator activity GO:0030234	180 (1.69)	221 (1.84)	Serine-type endopeptidase inhibitor activity GO:0004867 (CS:33; OV:67)
Metallochaperone activity GO:0016530	1 (0.01)		Copper chaperone activity GO:0016531 (CS:1)
Molecular transducer activity GO:0060089	111 (1.04)	124 (1.03)	Signal transducer activity GO:0004871 (CS:38; OV:49)
Nutrient reservoir activity GO:0045735	2 (0.02)	2 (0.02)	Nutrient reservoir activity GO:0045735 (CS:2; OV:2)
Proteasome regulator activity GO:0010860	2 (0.02)	1 (0.01)	Proteasome activator activity GO:0008538 (CS:2; OV:1)
Structural molecule activity GO:0005198	218 (2.05)	224 (1.86)	Structural constituent of ribosome GO:0003735 (CS:132; OV:145)
Transcription regulator activity GO:0030528	197 (1.85)	229 (1.90)	Transcription factor activity GO:0003700 (CS:99; OV:121)
Translation regulator activity GO:0045182	29 (0.27)	31 (0.26)	Translation initiation factor activity GO:0003743 (CS:17, OV:22)
Transporter activity GO:0005215	489 (4.60)	607 (5.04)	Atpase activity, coupled to transmembrane movement of ions, phosphorylative mechanism GO:0015662 (CS:48; OV:63)

a Values in parentheses are the percentage of the total number of predicted proteins within each GO category (i.e. biological process, molecular function or cellular component).

b The most frequently reported GO category and number of sequences within each category are summarized for each parental GO category.

The parental (i.e. level 2) and specific GO categories were assigned according to InterPro domains with homology to functionally annotated genes.

Significant similarity between protein sequences predicted for each *C. sinensis* and *O. viverrini* and those in the KOBAS database allowed 9,847 and 11,092 sequences to be assigned to 242 and 249 standardized (KEGG) biological pathway terms, respectively (Table 2). Like the functional annotation inferred using GO terms, biological pathways were similarly represented for the two transcriptomic data sets (Table 7). A significant proportion of molecules was associated with carbohydrate (7–9%) or amino acid (8%) metabolism, in agreement with the results of the GO-based analysis (Table 6). Cellular processing pathways were also frequently identified, including those associated with signal transduction (11–12%), cell communication (6–7%) and the endocrine (7–8%) and immune (4–5%) systems (Table 7). Importantly, 7–8% of predicted proteins from the two Asian liver flukes were linked to biological pathways that, when perturbed, can result in the development of cancer in humans (see Table 7), including molecules similar to integrins, regulatory GTPases, tyrosine and serine/threonine kinases and growth factors [63], [64].

**Table 7:** Summary of biological key pathways predicted from amino acid sequences encoded in the transcriptome of the adult stage of each *Clonorchis sinensis* and *Opisthorchis viverrini* based on homology to annotated proteins in the Kyoto Encyclopedia of Genes and Genomes (KEGG) biological pathways database.

Parent KEGG pathway	*C. sinensis* sequences (%)^a^	*O. viverrini* sequences (%)	Top KEGG pathway term^b^
Cellular processes			
Behavior	1 (0.01)	3 (0.04)	Circadian rhythm ko04710 (CS:1; OV:3)
Cell communication	471 (6.54)	507 (6.29)	Focal adhesion ko04510 (CS:168; OV:174)
Cell growth and death	249 (3.46)	287 (3.56)	Cell cycle ko04110 (CS:109; OV:123)
Cell motility	137 (1.90)	135 (1.67)	Regulation of actin cytoskeleton ko04810 (CS:137; OV:135)
Development	120 (1.67)	116 (1.44)	Axon guidance ko04360 (CS:96; OV:94)
Endocrine system	508 (7.06)	603 (7.48)	Insulin signaling pathway ko04910 (CS:151; OV:167)
Immune system	284 (3.95)	402 (4.99)	Leukocyte transendothelial migration ko04670 (CS:64; OV:70)
Nervous system	150 (2.08)	180 (2.23)	Long-term potentiation ko04720 (CS:88; OV:116)
Sensory system	46 (0.64)	76 (0.94)	Olfactory transduction ko04740 (CS:31; OV:55)
Environmental information processing			
Membrane transport	116 (1.61)	132 (1.64)	Other ion-coupled transporters ko00000 (CS:45; OV:48)
Signaling molecules and interaction	103 (1.43)	115 (1.43)	Neuroactive ligand-receptor interaction ko04080 (CS:37; OV:46)
Signal transduction	794 (11.03)	957 (11.87)	MAPK signaling pathway ko04010 (CS:162; OV:194)
Genetic information processing			
Folding, sorting and degradation	281 (3.90)	308 (3.82)	Ubiquitin mediated proteolysis ko04120 (CS:132; OV:153)
Replication and repair	106 (1.47)	101 (1.25)	Other replication, recombination and repair proteins ko00000 (CS:50; OV:51)
Transcription	87 (1.21)	89 (1.10)	RNA polymerase ko03020 (CS:41; OV:34)
Translation	192 (2.67)	243 (3.01)	Ribosome ko03010 (CS:91; OV:104)
Human diseases			
Cancers	547 (7.60)	580 (7.20)	Colorectal cancer ko05210 (CS:71; OV:73)
Infectious diseases	37 (0.51)	46 (0.57)	Epithelial cell signaling in *Helicobacter pylori* infection ko05120 (CS:37; OV:46)
Metabolic disorders	33 (0.46)	38 (0.47)	Type II diabetes mellitus ko04930 (CS:22; OV:25)
Neurodegenerative disorders	102 (1.42)	123 (1.53)	Huntington's disease ko05040 (CS:40; OV:46)
Metabolism			
Amino acid metabolism	565 (7.85)	659 (8.18)	Lysine degradation ko00310 (CS:75; OV:79)
Biosynthesis of polyketides and nonribosomal peptides	1 (0.01)	2 (0.02)	Biosynthesis of ansamycins ko01051 (CS:1; OV:2)
Biosynthesis of Secondary Metabolites	114 (1.58)	124 (1.54)	Limonene and pinene degradation ko00903 (CS:31; OV:37)
Carbohydrate metabolism	623 (8.66)	598 (7.42)	Starch and sucrose metabolism ko00500 (CS:91; OV:97)
Energy metabolism	198 (2.75)	232 (2.88)	Oxidative phosphorylation ko00190 (CS:95; OV:104)
Glycan biosynthesis and metabolism	211 (2.93)	213 (2.64)	N-Glycan biosynthesis ko00510 (CS:55;OV:21)
Lipid metabolism	389 (5.40)	439 (5.45)	Glycerophospholipid metabolism ko00564 (CS:65; OV:80)
Metabolism of cofactors and vitamins	225 (3.13)	229 (2.84)	Folate biosynthesis ko00790 (CS:67; OV:67)
Metabolism of other amino acids	114 (1.58)	116 (1.44)	Selenoamino acid metabolism ko00450 (CS:33; OV:30)
Nucleotide metabolism	189 (2.63)	184 (2.28)	Purine metabolism ko00230 (CS:126; OV:121)
Xenobiotics biodegradation and metabolism	205 (2.85)	223 (2.77)	Benzoate degradation via CoA ligation ko00632 (CS:33; OV:41)

a Values in parentheses are the percentage of the total number of predicted proteins within each KEGG category.

b The most frequently reported KEGG biological pathway and number of sequences within each pathway.

**Figure 2:**
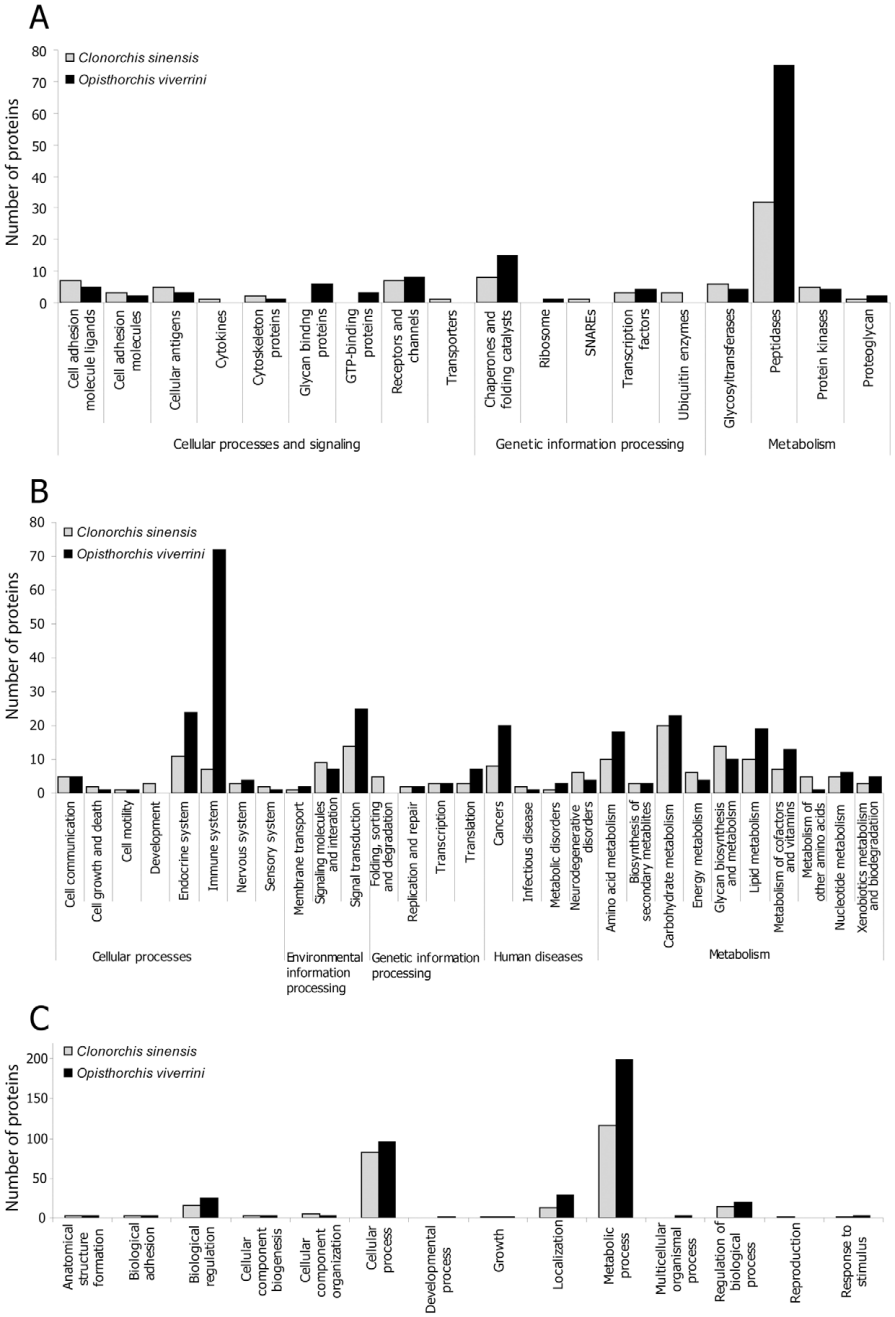
Characterization of the putative excretory/secretory proteins of the adult stage of each *Clonorchis sinensis* and *Opisthorchis viverrini*. Protein families (A) and biological pathways (B) were assigned to proteins based on their homology to annotated proteins in the Kyoto Encyclopedia of Genes and Genomes (KEGG) biological pathways. Within gene ontology (GO) categories, the parental (i.e. level 2) biological processes (C) were assigned to proteins according to InterPro domains with homology to functionally annotated genes. Individual KEGG and GO categories can have multiple mappings.

Proteins inferred from the *C. sinensis* and *O. viverrini* transcriptomes were screened for signal peptides and transmembrane domains. ORF-enriched sequences predicted to encode signal peptides (3,305–4,246 sequences; 6.5–6.9%) and transmembrane motifs (3,453–4,382; 6.8–7.1%) were identified (Table 2). Based on the presence of signal peptide domains, the absence of transmembrane domains and homology to known signal peptide domains, putative ES proteins (1,143–1,470; 2.3–2.4%) were identified in each data set (Table 2). Functionality was predicted for putative ES proteins by assigning them to standardized (KEGG) protein families, biological pathways and GO-inferred biological processes (Fig. 2). The majority of these molecules were predicted to be (i) metabolic proteins (99–150; 8.7–10.0%), such as peptidases, glycosyltransferases and protein kinases; (ii) genetic information processing proteins (15–20; 1.3–1.4%), such as chaperones, folding catalysts and transcription factors; and, (iii) cellular processes and signalling proteins (28–31; 2.1–2.4%), such as cell adhesion molecule ligands, cell adhesion molecules and cellular antigens (Fig. 2A). ES proteins (171–284; 15.0–19.3%) mapped to 29 parental KEGG pathways (Fig. 2B). ES protein sequences linked to the endocrine and immune systems were the predominant cellular processes inferred. Signal transduction and interaction molecules were mostly represented in environmental information processing pathways. ES proteins associated with metabolic pathways were predominantly linked to carbohydrate, lipid, amino acid or glycan metabolism. ES proteins inferred for *C. sinensis* and (particularly) *O. viverrini* were linked with biological pathways which are recognized or considered to be linked to carcinogenesis (<20 matches) in humans. These molecules include homologues to human proteins, such as calmodulin, c-Jun N-terminal kinase (JNK), laminin and the Ras family GTPase-Rap1, which, when deregulated, have tumorogenic potential [65]–[68]. ES proteins were also categorized according to biological processes inferred from homology with proteins for which GO information is available (259–382; 22.7–26.0%). Biological processes that were well represented included metabolic processes, biological regulation and localization (Fig. 2C).

## Discussion

The integrated genomic-bioinformatic approach used in the present study permitted a deep exploration of the transcriptomes of both *C. sinensis* and *O. viverrini*, with more than 50,000 unique sequences being identified for each species. More than 85% of the sequences generated from each of these transcriptomes (available *via* http://research.vet.unimelb.edu.au/gasserlab/index.html) were novel and thus represent a significant contribution to current databases [37]–[40], [45] and to scientific communities investigating parasites and neglected tropical diseases (NTDs). Based on similarity searches (BLASTx), more than 50% of the predicted protein sequences of *C. sinensis* and *O. viverrini* were inferred to be homologues, reflecting their relatively close biological [9] and phylogenetic [69] relationships. Amongst the trematodes *S. japonicum*, *S. mansoni* (Schistosomatidae), *F. hepatica* (Fasciolidae), *C. sinensis* and *O. viverrini* (Opisthorchiidae), the latter three species shared the greatest (29–31%) protein sequence homology. Interestingly, ∼70% of protein sequences in each of the data sets presented herein did not match those available in public databases and are interpreted to be specific to the biology of these liver flukes and their mode of existence in the hosts and, thus, could represent potential candidates for new drugs and/or vaccines. The considerable percentage of protein sequences predicted that have significantly greater similarity to those of mammals (20%) than nematodes (15%) is interesting, and in accordance with previous observations for parasitic trematodes [35], [36], [46]. This sequence similarity to mammalian molecules might reflect the capacity of the parasite to regulate host responses at the biochemical and immunological levels [36], [70]. However, since the free-living platyhelminth, *Schmidtea mediterranea*, also shares a high degree (60%) of sequence homology to the proteome of various vertebrates [71], several other factors might contribute to this observation, including the early evolutionary divergence of acoelomate platyhelminths (Lophotrochozoa) from higher invertebrates, such as parasitic nematodes (e.g., *Brugia*, Ecdysozoa) [72].

The present and future transcriptomic data sets, incorporating a wider array of free-living and parasitic invertebrates, should assist in identifying genes linked specifically to parasitism and also contribute significantly to our understanding of the evolution of the Metazoa. For each *C. sinensis* and *O. viverrini*, the numbers of sequences that clustered with genes from eukaryotic model organisms (including higher vertebrates, such as humans and mice, through to lower invertebrates, such as yeast) was approximately two-fold greater than expected. An estimate of the number of proteins expressed by an organism relates to the number of genes present as well as transcriptional variation [73]; thus, it is possible that alternative splice forms might have contributed to the high number of genes predicted from the ORF-enriched data sets. Other factors, such as a degree of heterozygosity within or among the individual worms used for sequencing, a higher representation of paralogous molecules than usual, multiple non-clustered ORFs spanning one large gene and/or sequencing errors within homopolymeric regions [52], [74]–[76], might also have contributed to this apparent overestimation. Independent data, generated using a short-read, deep-sequencing platform, such as the Illumina Genome Analyzer II [77], could be mapped to the present data sets to better define the complete transcriptomic profile and the number of genes for each species through enhanced assembly and annotation. Nonetheless, the present data sets for *C. sinensis* and *O. viverrini* are high quality drafts, and the assignment of molecules encoded in the transcriptomes to molecular functions and biological pathways reveals a substantial diversity of terms, comparable with those predicted for other parasitic trematodes, including *S. mansoni* (http://amigo.geneontology.org/; http://schistodb.net/schistodb20/)and *F. hepatica* [46].

The present transcriptomes inferred for *C. sinensis* and *O. viverrini* form a solid foundation and present unique opportunities for studying the developmental and reproductive biology of these parasites, parasite-host interactions and the pathogenesis of the diseases that these flukes cause in humans and animals. Importantly, the annotated data sets should also assist in the testing of current theories regarding the molecular basis of the pathogenesis of cholangiocarcinoma induced by chronic clonorchiasis or opisthorchiasis [24], [78], [79]. For instance, molecules excreted/secreted by the parasites are known to induce a proliferation of mammalian cells *in vitro* [29], [34] and have been suggested to play a role in the development of cholangiocarcinoma. Indeed, *O. viverrini* secretes a granulin-like molecule, which causes host cells to proliferate and is thought to be intimately involved in the initiation of carcinogenesis [80]. Clearly, the present transcriptomic data will be a valuable resource to support detailed proteomic analyses of extracellular molecules with mitogen-like activity to test and extend current hypotheses as to the role of *C. sinensis* and *O. viverrini* in the development of cholangiocarcinoma [24]. More broadly, the availability of the current transcriptomic data sets will substantially enhance the identification of both somatic and excretory/secretory (ES) and tegumenal proteins from *C. sinensis* and *O. viverrini*, following mass spectrometric analyses [41], [45]. These data, together with transcriptomic data for *F. hepatica* [46], contribute to a growing resource and a significant foundation for future comparative genomic, proteomic, metabolomic and pathophysiological investigations of liver flukes and the diseases that they cause.

The transcriptomes defined herein represent the adult stage of each *C. sinensis* and *O. viverrini*. However, there are surprisingly few transcriptional data for other developmental stages, with only 419 ESTs available for the metacercarial stage of *C. sinensis* [38] and none for *O. viverrini*. Currently, there are no sequence data for the other developmental stages (including miracidium, sporocyst, redia, cercaria and immature fluke) of these parasites. Future studies should now focus on the differential expression of genes through the multiple free-living and parasitic life history stages. The transcriptional data for adult *C. sinensis* and *O. viverrini* will underpin the characterization of transcriptional profiles of this stage, utilizing next-generation sequencing, microarray and/or quantitative real-time PCR analyses, which will have important implications for understanding development, reproduction as well as parasite-host interactions at the molecular level. Importantly, elucidating the transcriptomes of both immature and adult flukes provides the prospect of exploring immunopathological changes as well as carcinogenesis in humans at different stages of clonorchiasis and opisthorchiasis [6], [81].

In conclusion, the present transcriptomic data will assist in fundamental molecular studies of development, reproduction and metabolic pathways by providing a foundation for new developments in functional genomics. Gene perturbation assays are available for *S. mansoni* and *F. hepatica* [82]–[86], which indicates potential for the functional characterization of a wide range of molecules encoded in the transcriptomes of members of the family Opisthorchiidae. In the near future, it might be possible to predict and then characterize the function of parasite genes by employing probablistic functional networking, gene silencing and/or transgenesis. Bridging the gap between genomics and phenomics could provide unique insights into, for example, cellular differentiation, developmentally regulated gene expression, reproductive processes, signal transduction pathways linked specifically to parasitism and parasite-host interactions. In addition, the transcriptomes characterized here could support the definition of molecular or biological markers for the early diagnosis of disease. Importantly, the present transcriptomic data sets will also be an essential and powerful resource for the future assembly of the nuclear genomes of *C. sinensis* and *O. viverrini* as well as the determination of gene structures, prediction of alternative transcript splicing and the characterization of regulatory elements. Clearly, the future annotation of the genomes of these two parasites should also provide a foundation for the prediction of drug targets, based on an improved understanding of global biochemical pathways as well as genetic interactions [87], [88]. The recent success in the comparative analysis of nuclear genomes to infer metabolic pathways in microbial organisms [89], [90] appears to be an intellectual precedent for genomic sequencing. Coupled to extensive transcriptomic data sets for different developmental stages, genomic sequence data will enable extensive fundamental explorations and could facilitate the development of gene silencing, DNA-mediated transformation for these parasites as well as gene expression profiling and large-scale proteomic studies. Such advances should provide a basis for the delivery of applied outcomes, including the development of novel drugs and/or vaccines against *C. sinensis* and/or *O. viverrini* as well as diagnostic tools, particularly for the early diagnosis of cholangiocarcinoma. Given possible adverse effects of praziquantel treatment [32] and the high risk of re-infection following treatment [24], an emphasis must be placed on finding new and innovative intervention strategies against clonorchiasis and opisthorchiasis.
